# A Survey on the Indications, Diagnostic Efficacy, and Safety of Fiberoptic Bronchoscopy in Tikur Anbessa Specialized Hospital

**DOI:** 10.4314/ejhs.v35i1.8S

**Published:** 2025-12

**Authors:** Seid Ahmed Hassen, Hanan Yusuf Ahmed, Dawit Kebede Huluka, Aschalew Worku Ashagre, Mahfuz Ahmed Ibrahim, Berhanu Moges Abera, Misgan Tadesse Admassie

**Affiliations:** 1 Addis Ababa University, Department of Internal Medicine; 2 Division of Pulmonary and Critical care Medicine

**Keywords:** Bronchoscopy, usage, safety, diagnostic yield, Tikur Anbessa

## Abstract

**Background:**

Fiberoptic bronchoscopy is an essential diagnostic tool for infectious diseases such as tuberculosis and non-infectious conditions, including lung cancer, allowing direct visualization of the airways and tissue sampling. Given the high morbidity and mortality associated with these diseases, a comprehensive analysis of bronchoscopy utilization, safety, and diagnostic yield in Ethiopia is crucial. This study evaluated the indications, diagnostic efficacy, and safety of bronchoscopy over a two-year period.

**Methods:**

A retrospective analysis of electronic medical records, bronchoscopy procedural reports, and pathology and microbiology results from Tikur Anbessa Specialized Hospital was conducted. Data on demographics, smoking history, platelet count, coagulation profile, indications for the procedure, bronchoscopic interventions, and safety outcomes were collected. Data were analyzed using SPSS version 26 after ensuring completeness and consistency.

**Results:**

Of the 227 bronchoscopies performed, 205 records were reviewed. The mean patient age was 43.6 years. Most patients were male (52.2%), and 83.9% were aged 18–65 years. The most common indications for bronchoscopy were suspected malignancy (45.9%), tuberculosis (28.3%), and airway inspection (13.7%). The overall diagnostic yield was 75.6%. The diagnostic yield for Tuberculosis and lung cancer were 41.4% and 51.1% respectively. Adenocarcinoma was the most common lung cancer subtype (43.3%). Complications occurred in 6.8% of cases, with minor bleeding, hypertension, and pneumothorax reported rarely.

**Conclusion:**

Fiberoptic bronchoscopy at Tikur Anbessa Specialized Hospital is a safe and effective procedure with a high diagnostic yield, particularly for malignancy and tuberculosis. Although rare, pneumothorax may occur following blind transbronchial biopsy.

## Introduction

Respiratory diseases, particularly lung cancer, constitute a significant global health challenge, demanding precise and efficient diagnostic approaches. The development of fiberoptic bronchoscopy (FOB) in 1968 changed the practice of pulmonary medicine more than any other technological advance. Unlike rigid bronchoscopy, FOB is safer, easier to perform, and can be conducted on an outpatient basis using local anesthesia and conscious sedation, eliminating the need for general anesthesia. Despite its widespread use, diagnostic practices related to FOB remain inconsistent, even in developed countries such as the UK. The British Thoracic Society's 2013 guidelines recommend regular clinical audits of FOB procedures to assess diagnostic yield, patient satisfaction, and complications. Tikur Anbessa Specialized Hospital (TASH) in Ethiopia frequently uses FOB for diagnosing respiratory conditions. However, there has been no comprehensive evaluation of FOB's diagnostic yield and safety at TASH in the past decade. This study aims to assess the current use, safety, and diagnostic effectiveness of FOB at TASH from December 2021 to November 2023.(1–3)

One of the leading medical facilities in Ethiopia, Tikur Anbessa Specialized Hospital (TASH), frequently uses fiberoptic bronchoscopy as a diagnostic technique for respiratory disorders. However, over the past decade, a thorough assessment of its diagnostic yield and usefulness within the specific healthcare setting of TASH has not been systematically conducted. Therefore, the goal of this study is to provide an updated evaluation of its overall usage and safety and to assess its diagnostic yield at TASH, specifically from December 2021 to November 2023.

Despite the widespread use of FOB for diagnosing respiratory disorders, there has been little recent analysis of its effectiveness in Ethiopia's healthcare context, particularly at TASH. This study addresses a critical gap in knowledge regarding the diagnostic efficacy of FOB at TASH, which is essential for improving patient care. By reviewing FOB procedures conducted over the past two years, the study aims to identify areas for improvement and provide valuable insights into how the hospital can better manage pulmonary conditions.

The limited number of studies on FOB in Ethiopia—most recently conducted over a decade ago, with another dating back four decades—highlights the lack of updated data on its usage, safety, and diagnostic yield. The current study is important for several reasons. (4, 5) It fills a significant knowledge gap by evaluating the diagnostic efficacy of FOB at TASH. Overall, this research is expected to enhance the quality of respiratory care at TASH and has broader implications for improving diagnostic practices for respiratory diseases worldwide.

Reviewed studies on flexible bronchoscopy (FOB) across different countries provide insight into its diagnostic value and utility in managing pulmonary diseases. In India, bronchoscopy was particularly useful for diagnosing TB and lung malignancies, with a 62% diagnostic success rate, especially for TB and pneumonia cases. In Saudi Arabia, FOB proved effective in diagnosing pulmonary TB, lung cancer, and other conditions, with a low complication rate (1.5%), highlighting its safety. Studies in Pakistan and Norway reported similar success in diagnosing TB and malignancy, with minimal complications and diagnostic yields ranging from 46% to 51%. (6–10)

A multicenter study in the USA evaluated the diagnostic yield of fiberoptic bronchoscopy (FOB) for peripheral lesions and found an overall diagnostic yield of 53.7%, with lung cancer sensitivity ranging from 60% to 74%. Factors such as larger lesion size, non-upper lobe location, tobacco use, and transbronchial needle aspiration (TBNA) increased diagnostic yields, while advanced imaging techniques like electromagnetic navigation (EMN) were associated with lower yields. Complications occurred in 2.2% of cases, including pneumothorax and bleeding. Another study in China compared various bronchoscopic techniques for lung cancer diagnosis and found a higher diagnostic yield for biopsy (78%) compared with bronchial lavage (69%) and brushing (62%). In Singapore, FOB achieved a diagnostic yield of 71.2% for lung cancer, with higher yields for visible tumors and TBNA use. In France, modern techniques such as electromagnetic navigation bronchoscopy (ENB) and radial-probe EBUS yielded a diagnostic rate of 76%. Studies examining FOB safety in elderly patients and those with comorbidities found it generally safe, even in patients aged 85 years and older. Additionally, reviews of bronchoscopies performed in COVID-19 patients highlighted its diagnostic and therapeutic value, demonstrating that, with proper precautions, FOB remains safe and effective. Overall, FOB is a valuable diagnostic tool across diverse patient populations, although its use remains limited in developing countries. (11–18)

African studies from South Africa, Nigeria, Ghana, and Tanzania demonstrate FOB's value in diagnosing lung cancer, TB, and other pulmonary conditions, although challenges such as limited resources and underutilization persist. In Ethiopia, earlier studies reported a low diagnostic yield (21%) due to resource limitations, while more recent studies showed improvement (48%) with combined diagnostic techniques. A study from the USA further emphasized that lesion size and location influence FOB's diagnostic yield, which was higher for central lesions (82%) than peripheral lesions (53%). Collectively, these findings underscore FOB's role as a valuable diagnostic tool for pulmonary diseases while highlighting the need for improved access, training, and resources, particularly in low-income settings. (19–24)

The study aims to assess the indications, diagnostic efficacy, and safety of fiberoptic bronchoscopy (FOB) at Tikur Anbessa Specialized Hospital from December 2021 to November 2023. Specific objectives include determining the number of FOB procedures performed, identifying indications for FOB, evaluating its diagnostic effectiveness in respiratory diseases, and analyzing associated complications and safety concerns.

## Methods and Materials

This retrospective cohort study was conducted at Tikur Anbessa Specialized Hospital in Addis Ababa, Ethiopia, and included patients aged 14 years and older who underwent fiberoptic bronchoscopy (FOB) between December 2021 and November 2023. The study aimed to assess FOB utilization, diagnostic efficacy, and complications. Variables included age, sex, smoking history, comorbidities, HIV status, bronchoscopic techniques, suspected diagnoses, platelet count, coagulation profile, tumor visibility, size, and location. Data collection was performed using structured questionnaires based on patient medical records and bronchoscopy reports, with pretesting conducted to ensure accuracy. Data were analyzed using SPSS software, and ethical clearance was obtained from the Institutional Review Board.

Tikur Anbessa Specialized Hospital (TASH), located in Addis Ababa, is the largest specialized hospital in Ethiopia and serves as a national referral center. The Pulmonary and Critical Care Division is staffed by eight senior consultants with specialization in Internal Medicine and subspecialty training in Pulmonary and Critical Care Medicine (PCCM). The division also includes two second year and four first-year PCCM fellows. Bronchoscopy is a core competency required for PCCM fellows prior to program completion. Procedures are conducted by senior fellows under consultant supervision and supported by two well-trained nurses.

The rationale for selecting the December 2021 to November 2023 study period is based on the implementation of the hospital's electronic medical record system five years ago, one year prior to the COVID-19 pandemic. During the pandemic period, bronchoscopy services experienced significant disruption. Including earlier periods may not have provided reliable real-time data. Additionally, given the recovery and stabilization phase of healthcare services following COVID-19, evaluating bronchoscopy during this period offers valuable insights into post-pandemic diagnostic practice adaptation.

**Inclusion criteria**: All patients aged ≥ 14 years who underwent FOB at Tikur Anbessa Specialized Hospital during the study period. Patients aged 14 years and older are managed in adult clinics and were therefore included.

**Exclusion criteria**: Patients who underwent FOB but whose bronchoscopy reports were completely unavailable, including cases registered in procedure logs but lacking retrievable EMR or hardcopy documentation of findings.

### The following Operational definitions were used in this study

**Diagnostic yield**: The proportion of bronchoscopies providing a definitive diagnosis based on histology, cytology, or microbiology among suspected cases, calculated as the number of correct bronchoscopic diagnoses divided by the total number of suspected cases undergoing bronchoscopy.

**Complication:** Any adverse event occurring during or immediately after bronchoscopy—including bleeding requiring intervention, pneumothorax, hypoxia, arrhythmia, infection, or hospitalization—documented in the patient record.

**Visible tumor**: Any intraluminal endobronchial mass or lesion directly visualized during bronchoscopy and documented by the bronchoscopist.

## Results

**Patient sociodemographic characteristics**: The study evaluated outcomes from 227 bronchoscopies conducted over two years, with complete records available for 205 cases. A total of 22 cases were excluded due to incomplete or lost results, of which 7 had completely missing records.

Among the 205 cases, 107 patients (52.2%) were male. The median age was 43 years (range: 14–81 years), with a mean age of 43.6 years. Most patients (63.4%) resided outside Addis Ababa (Table 1).

**Table 1:** Patient Socio-demographic characteristics

Variables	Male	Female	Total	Percent
Age in years				
<18	3	6	9	4.4
[18-30)	18	17	35	17.1
[30-40)	31	20	51	24.9
[40-50)	19	16	35	17.1
[50-60)	16	15	31	15.1
[60+	20	24	44	21.5
Subtotal	107	98	205	100
Address				
Addis Ababa	75			36.6
Other locations	130			63.4
Total	205			100

**Risk Factors and comorbidities**: HIV status was documented for 108 patients, of whom 10.2% tested positive. Smoking status was available for 177 patients, with 12.4% identified as smokers, half of whom were current smokers. Comorbid conditions included hypertension (14.2%), bronchiectasis (11.7%), diabetes mellitus (6.8%), asthma (5.9%), and tuberculosis (5.9%). Most patients (95.6%) had platelet counts above 50,000, and INR values were predominantly between 1.2 and 2 (Table 2).

**Table 2:** Disease risk factors and comorbid disease conditions

Variable	Frequency (%)
**Smoking condition**	
Unknown/Not Documented	28 (13.7)
Known smoking status	177 (86.4)
From those with known status	
(177)	
Yes	22 (12.4)
No	155 (87.6)
From those who are smokers (22)	
Current smokers	11 (50.0)
Ex-smokers	11 (50.0)
**INR Value**	
<1.2	59 (28.8)
1.2-2	105 (51.2)
2-3	3 (1.5)
not done	38 (18.5)
**Platelet count**	
<50,000	2 (1.0)
50,000-100,000	11 (5.6)
≥100,000	183 (93.4)
**Comorbid diseases**	
None	100 (48.8)
HTN	29 (14.2)
Bronchiectasis	24 (11.7)
DM	14 (6.8)
Asthma	12 (5.9)
HIV	12 (5.9)
TB	4 (2.0)
COPD	3 (1.6)
Others	51 (24.9)

**Indications for bronchoscopy**: The most common indication for bronchoscopy was suspected malignancy or mass (45.9%), followed by tuberculosis (28.3%) and airway inspection (13.7%). Additional indications included hemoptysis, sarcoidosis, recurrent pneumonia, and foreign body aspiration. Among 94 patients suspected of malignancy, 36.2% had visible tumors, most commonly located in the right upper lobe (Table 3).

**Table 3:** Bronchoscopy indications and gross findings

Variable	Frequency (%)
**Preprocedural consideration/Indication**	
Lung mass/suspected malignancy	94 (45.6)
TB	58 (28.3)
Other	18 (8.8)
Airway inspection	28 (13.7)
Hemoptysis	17 (8.3)
Sarcoidosis	11 (5.4)
Pneumonia/recurrent	7 (3.4)
Foreign body	6 (2.9)
**General Gross bronchoscopic findings**	
Mucosal abnormalities/	61 (29.8)
Inflammatory changes	
Normal	60 (29.3)
Endobronchial mass/tumor	34 (16.6)
Extrinsic compression/narrowed segment	29 (14.2)
LAPs (if it was EBUS)	13 (6.3)
increased secretion	19 (9.3)
Others	19 (9.3)

**Bronchoscopic findings**: The most frequent gross bronchoscopic findings were mucosal abnormalities and inflammatory changes (29.8%), followed by normal findings (29.3%). Endobronchial masses or tumors were identified in 16.6% of cases, while extrinsic compression or narrowed airway segments were noted in 14.2%. Among 19 EBUS procedures, lymphadenopathy was detected in 68.4% of cases. Other findings included increased secretions, bleeding, and foreign bodies.

**Safety of bronchoscopy**: Bronchoscopy demonstrated a favorable safety profile, with 93.2% of procedures performed without complications. Minor complications—including mild bleeding, hypertension, and hypoxemia—occurred in 5.9% of cases. Pneumothorax occurred in 1% of procedures. All complications were appropriately managed. Hypoxemia was treated with supplemental oxygen, hypertension with medication, and pneumothorax with chest tube insertion. These findings indicate that bronchoscopy is generally safe, though careful monitoring remains essential, particularly during invasive procedures such as biopsy.

**Samples collected and diagnostic modalities**: Samples collected included bronchoalveolar lavage (BAL), transbronchial biopsy (TBBx), endobronchial biopsy (EBBx), brush biopsy, and EBUS-guided samples. A total of 399 diagnostic tests were performed. GeneXpert (35.1%) and cytology (34.6%) were the most frequently utilized modalities, followed by pathology (19.3%), culture (5.8%), Gram stain (4.5%), and fungal stain (0.8%). This multimodal diagnostic approach reflects the complexity of pulmonary disease evaluation (Table 4).

**Table 4:** Samples obtained during the procedure and the corresponding tests requested*

Variable	Frequency (%)
**Sample taken during the procedure**	
BAL (Bronchoalveolar Lavage)	161 (78.5)
TBBx (Transbronchial Biopsy)	53 (25.9)
EBBx (Endobronchial Biopsy)	21 (10.2)
Brush Bx (Brush biopsy)	3 (1.5)
Blind TBNA (Transbronchial	5 (2.4)
Needle Aspiration)	
EBUS guided sampling	19 (9.3)
None	27 (13.2)
**Sample sent for tests**	
Pathology	77 (43.4)
GeneXpert	140 (28.3)
Cytology	138 (67.3)
Culture	23 (11.2)
Gram Stain	18 (8.8)
Fungal stain	3 (1.5)
None	27 (13.2)

* The percentage denotes the ratio of tests per total bronchoscopies conducted, i.e., 205)

**Final diagnosis and diagnostic yield**: Transbronchial biopsy results were positive for malignancy or granuloma in 22.9% of cases, while 54.7% were negative. Endobronchial biopsy demonstrated a higher diagnostic yield, with 71.4% positive for malignancy or granulomatous disease. EBUS-guided samples yielded granulomas (3 cases), malignancy (3 cases), suspicious results, and negative findings (3 cases), while three samples were lost. Final diagnoses showed lung cancer as the most common condition 48/205 (23.4%), followed by tuberculosis (14.6%). Other diagnoses included normal findings (24.4%), sarcoidosis (5.4%), bronchiectasis (2.9%), carcinoid tumors (2.4%), and aspergilloma (2%). From those 48 lung cancer cases, a total of 30 cases were confirmed only by FOB pathology and histology samples.

Adenocarcinoma was the most common lung cancer subtype (43.3%), followed by squamous cell carcinoma (20%) and small cell lung cancer (13.3%). Tuberculosis was confirmed in 24 of 58 suspected cases, while all sarcoidosis suspected cases were confirmed to be sarcoidosis (Tables 5 and 6).

**Table 5:** The final working diagnosis of patients who undergone FOB

Working Diagnosis	Frequency (%)
Normal	50 (24.4)
Lung cancer	48 (23.4)
TB	30 (14.6)
Other	31 (15.1)
Sarcoidosis	11(5.4)
Bronchiectasis	6 (2.9)
Carcinoid tumor	5 (2.4)
Tracheal stenosis	5 (2.4)
Aspergiloma	4 (2.0)
pneumonias (eosinophilic, necrotizing, HP) & lung abscess	15 (7.3)
Total	205 (100)

**Table 6:** Histologic types of diagnosed lung cancers

Type of lung ca.	Frequency (%)
Adenocarcinoma	13 (43.3)
SCC	6 (20.0)
SCLC	4(13.3)
NSCLC	5 (16.7)
Adenosquamous	1 (3.3)
Undifferentiated	1 (3.3)
Total	30 (100)

Overall, 155 positive diagnoses were identified among 205 cases, yielding an overall diagnostic rate of 75.6% (95% CI: 69.3–81.0). Pathology biopsy yielded a diagnostic rate of 39.1%. Cytology yielded a diagnostic rate of 11.6% for lung cancer, while the diagnostic yield for tuberculosis was 41.4%. Flexible bronchoscopy demonstrated diagnostic yields of 51.1% (48/94) for lung cancer and 41.4% for tuberculosis.

## Discussion

In this two-year retrospective survey, 205 FOB procedures performed at TASH were analyzed: 97 cases in 2022 and 108 cases in 2023. In this study, 52.2% of patients who underwent FOB were male, and the overall mean age was 43.6 years. This finding is comparable to studies from Kenya by Bashir et al., Nigeria by Adewole et al., Tanzania by Ndilanha et al., and Saudi Arabia by Qanash et al., where mean ages ranged from 51.3 years in Kenya to 54.8 years in Nigeria (8, 21, 23, 25).

The HIV serostatus was known for 52.7% of patients, of whom 10.2% were HIV-positive. In contrast, 74% of participants in a South African study by Ajayi et al. had known HIV serostatus, with 29% testing positive. This difference may reflect variations in HIV prevalence between the countries (19).

Despite the lack of evidence supporting routine determination of platelet count and coagulation profiles, 95.6% of cases had complete blood count (CBC) testing, and 81.46% had INR determination. A similar overutilization of INR testing was observed in Kenya by Bashir et al. (75.9%), while most other studies did not comment on this practice. However, the British Thoracic Society recommends these tests only when there is clinical suspicion that results may be abnormal (2, 25).

The most common indication for FOB in our study was suspected mass or malignancy, accounting for 45.9% of cases, followed by tuberculosis (28.3%) and airway inspection (13.7%). This finding is consistent with a study from Ghana by Issaka et al., which identified suspected lung cancer as the most common indication (48.5%), but lower than the Tanzanian study by Ndilanha et al., where 67.6% of indications were for lung cancer. It was slightly higher than the proportion reported in Kenya by Bashir et al. (35.7%). A possible explanation is that FOB may be used more frequently for lung cancer diagnosis in some settings compared to other respiratory disorders (22, 23, 25).

Pre-procedure sputum GeneXpert MTB/RIF testing was performed in 79.5% of cases, even when tuberculosis was not the primary consideration. While it may be reasonable to include tuberculosis in the differential diagnosis in countries such as ours, this practice may also contribute to test scarcity for other patients in need, as has recently been observed in our setting.

The overall complication rate in our study was 6.8% (14 cases), the majority of which (12 cases) were minor complications. Only two cases (1%) developed pneumothorax as a major complication, and no deaths were recorded. In comparison, the AQuIRE study from the USA reported a complication rate of 2.2%, with pneumothorax being the most common, while a Kenyan study reported a rate of 3.2%. A lower complication rate of 1.3% was reported in another US study by Pue et al., possibly because only a minority of patients underwent biopsy; among those who did, the complication rate was 6.8%. An older US study by Dreisin et al. in 1978 reported a significantly higher complication rate of 11%, including one death, likely reflecting differences in expertise and equipment compared to current practice. In our hospital, minor complications were managed promptly at the bronchoscopy table, while the two patients who developed pneumothorax required brief emergency room admission followed by timely and successful management. Notably, both pneumothorax cases occurred following blind transbronchial biopsy, highlighting the potential benefit of ultrasound guidance or fluoroscopy. Overall, complication rates depend on the type of sampling performed and whether late complications are included (12, 25–27).

The overall diagnostic yield of FOB in our study was 75.6%, which is identical to an unpublished study conducted at our hospital 10 years ago by Fahmi et al. (75%). This yield is significantly higher than that reported by Teklu B. four decades ago (21%) and the Nigerian study by Adewole et al. (62%). Our findings are comparable to studies from Egypt (78.6%) and Kenya by Bashir et al. (81.9%). The diagnostic yield for malignancy in cases with visible endobronchial tumors was 73.68%, which falls short of the British Thoracic Society's recommended target of 85% but remains close and comparable to results from Singapore by Liam et al. (83.2%) and Kenya by Bashir et al. (87.2%). This yield was higher than that reported by Adewole et al. (54.5%) but lower than findings from Egypt by Mohamed et al. (94.7%) and Halima et al. (95%), indicating room for further improvement (2, 14,21,24, 25,28).

Cytology demonstrated the lowest diagnostic yield for lung cancer, with only 16 of 138 samples testing positive for malignancy (11.6%), and an additional 11.6% reported as suspicious. Of 322 fluid samples, 17 results were lost; among the remaining 305 samples, only 42 were positive, yielding a diagnostic rate of 13.8% (42/305). Similarly low diagnostic yields from fluid samples have been reported in Tanzania by Ndilanha et al. (9.6%) and India by Roth et al. (6.9%). However, this yield was markedly lower than that reported in Spain by de Gracia et al. (33%), possibly due to the higher lavage volume used (100 mL). Additional factors contributing to the low yield in our study may include inadequate wedging of the bronchoscope into the affected bronchus and the relatively small lavage volumes obtained (10-20 mL)(10, 23,29).

Adenocarcinoma was the most prevalent histologic subtype, accounting for 43.3% of cases, similar to findings from Tanzania (Ndilanha et al.), France (Mhanna et al.), and Kenya (Bashir et al.), where adenocarcinoma accounted for 33.9%, 54%, and 45%, respectively. In contrast, studies from India (Kshatria et al.), Egypt (Halima et al.), and Saudi Arabia (Alamoudi et al.) reported squamous cell carcinoma as the most common subtype, accounting for 44%, 34.2%, and 61%, respectively. In these studies, adenocarcinoma accounted for 24% in India and 17% in Egypt, while small cell carcinoma was the second most common subtype in Saudi Arabia (22%). These differences are likely related to smoking prevalence, with approximately half of patients in Egypt and Saudi Arabia being smokers compared to only 12.4% in our study, representing a four-fold difference. This supports the established association between smoking and squamous cell and small cell carcinomas (7, 15, 23, 25, 28).

The proportion of smokers (current and former) among patients with malignancy was 31.3% (15/48), similar to the Kenyan study by Bashir et al. (36.3%) but substantially lower than the Saudi Arabian study by Alamoudi et al. (84%). This difference likely reflects the much higher smoking prevalence in the Saudi study population. Among smokers in our study, 68.2% (15/22) were diagnosed with lung cancer, with squamous cell carcinoma being the most common subtype. Notably, 68.8% (23/48) of lung cancer patients in our study were never smokers, suggesting that factors other than smoking play a significant role in lung cancer development in this population.

Of the 399 tests performed (77 pathology samples and 322 fluid samples), 25 results were missing (8 pathology and 17 fluid samples), resulting in a loss rate of 6.3% (25/399). A similar loss rate was reported in Tanzania, where 7.7% (35/451) of test results were missing (23).

Due to its retrospective nature, this study relied on existing medical records, resulting in missing data, particularly regarding smoking status, which limited reliable assessment of the correlation between smoking and lung cancer. Some pathology reports were also incomplete. Additionally, as a single-center study conducted at Tikur Anbessa Specialized Hospital, the findings may not be generalizable to other settings. Diagnostic yield, usage, and safety may vary depending on bronchoscopist expertise, and potential confounding factors—such as physician-assessed pre-test probability of malignancy—were not consistently documented.

Our study demonstrates that the most common indication for FOB at TASH is the evaluation of suspected masses. FOB at TASH shows a higher diagnostic yield compared to other African reports, with the highest yield observed in cases with visible tumors and endobronchial sampling. Cytology from fluid analysis yielded the lowest diagnostic rate for malignancy. FOB practice at TASH is safe; however, although rare, blind transbronchial biopsy carries a risk of pneumothorax. Adenocarcinoma was the most common lung cancer subtype overall, while squamous cell carcinoma predominated among smokers.

FOB indications could be expanded to include therapeutic interventions for advanced malignancies. Efforts are also needed to improve the diagnostic yield of cytology from fluid samples to enable less invasive lung cancer diagnosis. Using brush biopsy alongside other sampling techniques may further enhance yield. Collaboration with pathologists and optimization of sample handling and processing may improve cytologic results and partially explain the lower yield compared with studies from Egypt.

The utilization of fungal studies from FOB samples remains very low in a referral hospital managing a high volume of hematologic and solid malignancies. Measures should be implemented to minimize sample loss during pathological examination, emphasizing adequate sample acquisition and improved communication with pathologists to enhance diagnostic yield. Given the demonstrated safety and efficacy of FOB, its use is recommended for any patient who may benefit from the procedure.
